# Low SP1 Expression Differentially Affects Intestinal-Type Compared with Diffuse-Type Gastric Adenocarcinoma

**DOI:** 10.1371/journal.pone.0055522

**Published:** 2013-02-20

**Authors:** Hun Seok Lee, Cheol-Keun Park, Ensel Oh, Özgür Cem Erkin, Hun Soon Jung, Mi-Hyun Cho, Mi Jeong Kwon, Seoung Wan Chae, Seok-Hyung Kim, Li-Hui Wang, Min-Jeong Park, Su-Yeon Lee, Ho Bin Yang, Lina Jia, Yoon-La Choi, Young Kee Shin

**Affiliations:** 1 Research Institute of Pharmaceutical Science, College of Pharmacy, Seoul National University, Seoul, Korea; 2 Department of Pathology, Samsung Medical Center, Sungkyunkwan University School of Medicine, Seoul, Korea; 3 College of Pathology, Kyungpook National University, Deagu, Korea; 4 Department of Pathology, Kangbuk Samsung Hospital, Sungkyunkwan University School of Medicine, Seoul, Korea; 5 Department of Pharmacology, College of Pharmacy, Shenyang Pharmaceutical University, Shenyang, China; Okayama University, Japan

## Abstract

Specificity protein 1 (SP1) is an essential transcription factor that regulates multiple cancer-related genes. Because aberrant expression of SP1 is related to cancer development and progression, we focused on SP1 expression in gastric carcinoma and its correlation with disease outcomes. Although patient survival decreased as SP1 expression increased (P<0.05) in diffuse-type gastric cancer, the lack of SP1 expression in intestinal-type gastric cancer was significantly correlated with poor survival (P<0.05). The knockdown of SP1 in a high SP1-expressing intestinal-type gastric cell line, MKN28, increased migration and invasion but decreased proliferation. Microarray data in *SP1* siRNA-transfected MKN28 revealed that the genes inhibiting migration were downregulated, whereas the genes negatively facilitating proliferation were increased. However, both migration and invasion were decreased by forced SP1 expression in a low SP1-expressing intestinal-type gastric cell line, AGS. Unlike the intestinal-type, in a high SP1-expressing diffuse-type gastric cell line, SNU484, migration and invasion were decreased by *SP1* siRNA. In contrast to previous studies that did not identify differences between the 2 histological types, our results reveal that low expression of SP1 is involved in cancer progression and metastasis and differentially affects intestinal-type compared with diffuse-type gastric adenocarcinoma.

## Introduction

Lauren's classification, which divides gastric carcinoma into intestinal and diffuse subtypes according to tumor morphological features, has been adopted worldwide [1]. Each tumor type develops through a distinct carcinogenic pathway [2]. The 2 histological types have unique macroscopic appearances reflecting the difference in their microscopic growth patterns. In the intestinal-type carcinoma, the macroscopic margins correspond approximately to the microscopic spread, whereas the poor differentiation of the diffuse-type carcinoma allows its submucosal extension far beyond its macroscopic borders [3]. This difference in tumor spread is of clinical importance in choosing an appropriate treatment [1].

SP1 (specificity protein 1) is a sequence-specific DNA-binding protein that is involved in the activation and regulation of cellular transcription. SP1 is an essential transcription factor for many genes [4] whose abnormal function may result in tumor cell development, differentiation, and proliferation in various types of cancers, including breast and pancreatic [5], [6]. Nevertheless, the role of SP1 in gastric carcinoma has not been clarified, although a line of evidence clearly emphasizes its clinical importance; SP1 overexpression is reportedly correlated with angiogenic potential and poor prognosis in human gastric carcinoma [7]–[9].

We examined SP1 expression and the clinical significance of its relationship with Lauren's histological classification of the different types of gastric cancer tissue samples and cell lines. We suggest that SP1 may have different functions in intestinal- and diffuse-type gastric carcinomas.

## Materials and Methods

### Patient tissues and immunohistochemical analysis

Using 396 gastric tissue samples, including 268 carcinomas, 16 high-grade dysplasias, 12 low-grade dysplasias, 38 intestinal metaplasias, 24 chronic atrophic gastritis samples, and 38 normal gastric epithelium samples from Chungbuk National University Hospital (Cheongju, Korea) and Samsung Medical Center (Seoul, Korea), a tissue microarray with 3-mm diameter tissue columns was constructed. SP1 immunostaining was performed using a rabbit anti-SP1 antibody (1:50 dilution, Santa Cruz Biotechnology, Santa Cruz, CA), as previously reported [10]. The staining intensity and proportion of positively stained epithelial cells were evaluated, and an immunoreactive score (IS) for each sample was generated, as previously described [11]. These samples were divided into 2 categories based on the SP1 expression levels exhibiting maximal statistical significance in both intestinal- and diffuse-type gastric cancer: low (IS <2) and high (≥2).

### Cell lines and western blot analysis

Fifteen gastric cancer cell lines were maintained in appropriate culture media. Cells were harvested, and proteins were extracted for western blot analysis using primary antibodies against SP1, SP3, SP4, VEGF (vascular endothelial growth factor), CDH1 (E-cadherin), GAPDH (glyceraldehyde 3-phosphate dehydrogenase), and ACTB (beta-actin).

### Cell proliferation, migration and invasion assay

Control or *SP1*-specific siRNA (Thermo Scientific Dharmacon Products, Lafayette, CO) was transfected into SNU484 or MKN28 cells. The effects of *SP1* knockdown on cell proliferation were measured by the Water-Soluble Tetrazolium salts (WST) method (EZ-Cytox kit; Daeil Lab Service, Seoul, Korea). The cell viability was reported as a percentage of control siRNA-transfected cells.

To evaluate changes in cell migration and invasion, 3×10^4^ *SP1* siRNA- or *SP1*-plasmid-transfected cells were utilized in a CHEMICON QCM™ 24-well Cell Migration and Invasion Assay System (Millipore Corporation, Billerica, MA). Cell numbers were detected fluorometrically with a GENios reader (Tecan, Männedorf, Switzerland) and 485/535 nm filter set. The migration and invasion assays were performed in triplicate in at least 3 independent experiments. The values are expressed as the percentages compared with the controls.

### RNA preparation and qRT-PCR

Total RNA was extracted using TRIzol (Invitrogen, Grand Island, NY) and reverse transcribed to cDNA using the Superscript™ II First-Strand Synthesis System (Invitrogen). Following cDNA synthesis, qRT-PCR was performed as described [12] using primers for *SP1, VEGF, CDH1, CXCL10 (C-X-C motif chemokine 10), ACVRL1 (activin A receptor type II-like 1), CDKN2D (cyclin-dependent kinase inhibitor 2D), BCL2 (B-cell CLL/lymphoma 2)*, and *TRIB1 (tribbles homolog 1)*; *HPRT1 (Hypoxanthine-guanine phosphoribosyltransferase)* was used to normalize gene expression. The primer and probe sequences (Roche Diagnostics, Mannheim, Germany) are listed in Table S1.

### Gene expression array

Total RNA was extracted from control- or *SP1* siRNA-treated MKN28 cells using the RNeasy Kit (Qiagen, Valencia, CA). The RNA quality was assessed using an Agilent 2100 Bioanalyzer (Agilent Technologies, Palo Alto, CA). For microarray hybridization, 100 ng RNA from each sample was amplified and labeled with the Cy3 or Cy5 fluorescent dye. The quantity and purity of the fluorescently labeled cRNA were evaluated using a NanoDrop ND-1000 UV-Vis Spectrophotometer (Thermo Fisher Scientific, Wilmington, DE). Equal amounts of Cy3 and Cy5-labeled cRNA were hybridized to an Agilent Human Microarray (G4112F) for 17 h at 65°C. Two replicates with dye-swap were performed, and the hybridized microarrays were washed and scanned using an Agilent G2565BA scanner. Data were extracted from the scanned images using Agilent Feature Extraction. The expression data were analyzed using the open-source statistical language R (version 2.10.0) with the samr and GOstats packages.

### Statistical analysis

The association of SP1 expression levels with clinicopathological factors was assessed by Pearson's Chi-squared contingency table tests. The survival probabilities of two groups of SP1 were estimated by Kaplan-Meier curves and compared using the Log rank test. The Cox proportional hazard model was used to estimate the hazard ratios of various clinicopathological factors and SP1 expression on the survival of the gastric cancer patients. The survival analyses were performed using the open-source statistical language R (version 2.10.0) with the survival package.

For details, see Materials and Methods S1 (supporting information).

## Results

### Expression of SP1 through gastric tumorigenesis

**Figure 1:**
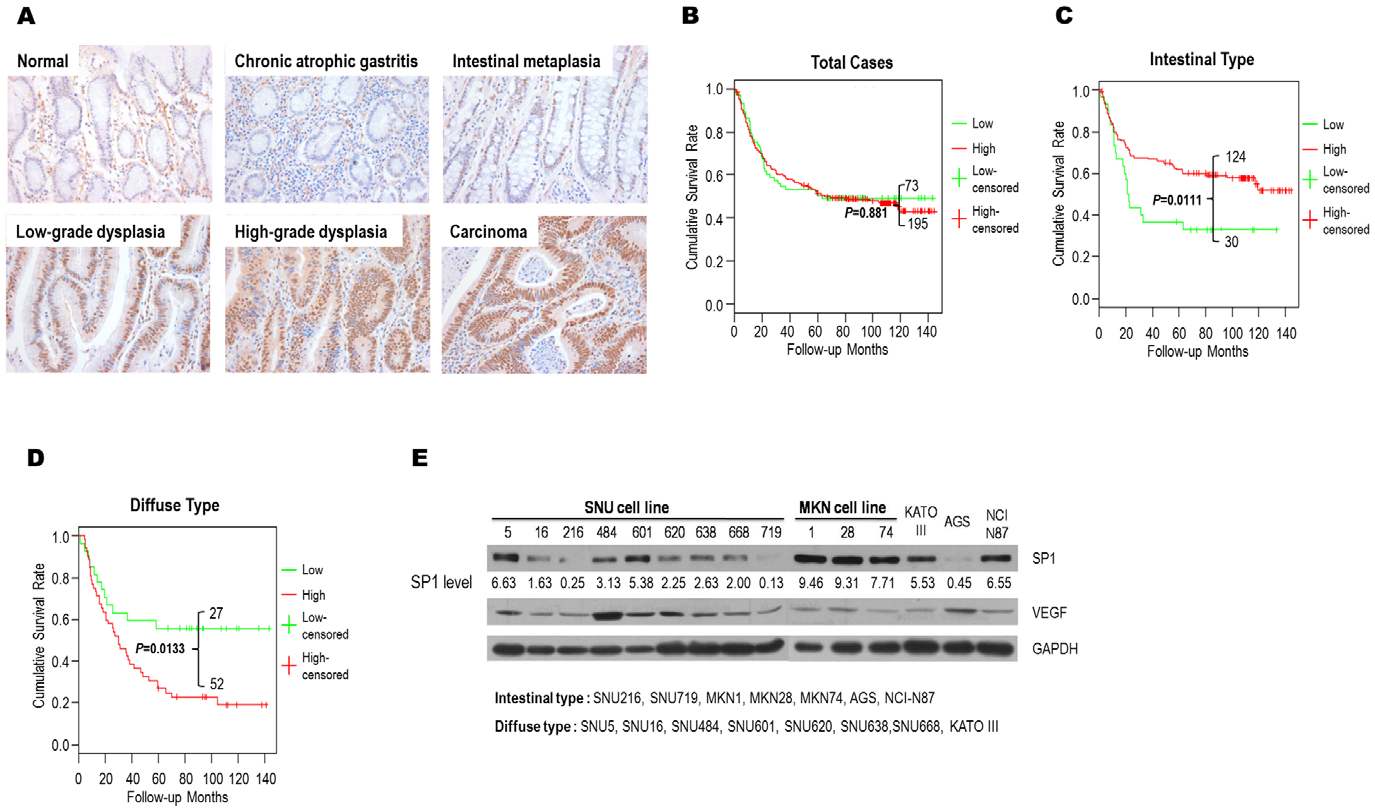
Immunohistochemical and survival analysis of SP1 expression in gastric cancer patients and SP1 expression in gastric cancer cell lines. A) The expression of SP1 increases with tumor progression from precancerous to malignant lesions. Positive staining is determined by a reddish-brown precipitate in the nuclei. B) The Kaplan-Meier survival probability with respect to SP1 expression levels in gastric carcinomas. Log-rank tests were performed to compare the survival of SP1 subgroups both within and between subtypes. C) The Kaplan-Meier survival probability with respect to SP1 expression levels in intestinal-type gastric carcinomas. Log-rank tests were performed to compare the survival of SP1 subgroups both within and between subtypes. *P*<0.05 was considered to be statistically significant. D) The Kaplan-Meier survival probability with respect to SP1 expression levels in diffuse-type gastric carcinomas. Log-rank tests were performed to compare the survival of SP1 subgroups both within and between subtypes. *P*<0.05 was considered to be statistically significant. E) The SP1 expression in 15 gastric cancer cell lines was measured by western blot analysis. The numbers indicate semi-quantified protein expression based on scanning densitometry normalized to GAPDH (a control). VEGF was also measured to compare its expression with SP1 in each cell type.

The immunochemical analysis of SP1 revealed a significant increase in its expression during tumor progression (p<0.0001) (Table S2). In normal and chronic atrophic gastritis cases, SP1 expression was low in 89.5% and 83.3% of the samples, respectively. In intestinal metaplasia, the frequency of high SP1 expression increased to 31.6%, and the proportion of cases exhibiting high SP1 expression increased further to 50.0% and 56.3% in low- and high-grade dysplasia, respectively. Moreover, as the tumor advanced to carcinoma, high SP1 expression was frequently observed (72.8%). SP1 expression therefore increases as normal gastric tissues develop into cancer (Figure 1A).

### Different patterns of SP1 expression and survival analyses in intestinal- and diffuse-type gastric cancer

Chi-square tests did not identify a significant association between SP1 expression levels and any clinicopathological features other than WHO classification and Lauren's histology type (*P*<0.05, Table 1). Moreover, a total patient survival rate based on Kaplan-Meier analysis have not shown significance (Figure 1B). Given that Lauren's histological classification is significantly correlated with SP1 expression in gastric cancer, we divided the samples according to their histological type and analyzed their correlation with clinicopathological features as well as their relationship with overall patient survival. Indeed, Kaplan-Meier survival analysis of these 2 groups identified different patterns (Figure 1C, D).

**Table 1:** Clinicopathological characteristics of gastric cancer patients and the correlation with SP1 expression.

Variables	Total			Intestinal-type			Diffuse-type		
	No. of patients	SP1 Low (%)	SP1 High (%)	No. of patients	SP1 Low (%)	SP1 High (%)	No. of patients	SP1 Low (%)	SP1 High (%)
Age									
<60	148	44 (29.7)	104 (70.3)	67	15 (22.4)	52 (77.6)	56	20 (35.7)	36 (64.3)
≥60	120	29 (24.2)	91 (75.8)	87	15 (17.2)	72 (82.8)	23	7 (30.4)	16 (69.6)
		*P* = 0.379			*P* = 0.552			*P* = 0.851	
Sex									
F	105	30 (28.6)	75 (71.4)	54	12 (22.2)	42 (77.8)	41	14 (34.1)	27 (65.9)
M	163	43 (26.4)	120 (73.6)	100	18 (18.0)	82 (82.0)	38	13 (34.2)	25 (65.8)
		*P* = 0.800			*P* = 0.676			*P* = 0.817	
Tumor size (cm)									
<5.0	86	23 (26.7)	63 (73.3)	53	7 (13.2)	46 (86.8)	21	10 (47.6)	11 (52.4)
≥5.0	170	47 (27.6)	123 (72.4)	96	22 (22.9)	74 (77.1)	58	17 (29.3)	41 (70.7)
		*P* = 0.996			*P* = 0.224			*P* = 0.212	
Stage									
I	66	18 (27.3)	48 (72.9)	42	5 (11.9)	38 (88.1)	7	3 (42.9)	4 (57.1)
II	35	8 (22.9)	27 (77.1)	26	3 (11.5)	23 (88.5)	9	5 (55.6)	4 (44.4)
III	91	26 (28.6)	65 (71.4)	46	9 (19.6)	37 (80.4)	36	14 (38.9)	22 (61.1)
IV	75	21 (28.0)	54 (72.0)	39	13 (33.3)	26 (66.7)	26	5 (19.2)	21 (80.8)
		*P* = 0.932			*P* = 0.057			*P* = 0.175	
WHO classification									
Tubular, Well	31	5 (16.1)	26 (83.9)	30	5 (16.7)	25 (83.3)	1	0 (0.0)	1 (100.0)
Tubular, Moderately	91	21 (23.1)	70 (76.9)	88	21 (23.7)	67 (76.3)	2	0 (0.0)	2 (100.0)
Tubular, Poorly	91	21 (23.1)	70 (76.9)	30	1 (3.3)	29 (96.7)	54	19 (35.2)	35 (64.8)
Signet ring cell type	48	21 (43.8)	27 (56.2)	3	1 (33.3)	2 (66.7)	18	5 (27.8)	13 (72.2)
Mucinous type	6	5 (83.3)	1 (16.7)	3	2 (66.7)	1 (33.3)	3	3 (100.0)	0 (0.0)
		*P*<0.001			*P* = 0.028			*P* = 0.106	
Invasion Depth									
T1	49	14 (28.6)	35 (71.4)	31	3 (9.7)	28 (90.3)	3	2 (66.7)	1 (33.3)
T2	53	12 (22.6)	41 (77.4)	35	5 (14.3)	30 (85.7)	17	6 (35.3)	11 (64.7)
T3	122	34 (27.9)	88 (72.1)	64	15 (23.4)	49 (76.6)	46	15 (32.6)	31 (67.4)
T4	39	12 (30.8)	27 (69.2)	22	6 (27.3)	16 (72.7)	12	4 (33.3)	8 (66.7)
		*P* = 0.927			*P* = 0.257			*P* = 0.693	
Nodal metastasis									
Yes	183	51 (27.9)	132 (72.1)	101	23 (22.8)	78 (77.2)	65	22 (33.8)	43 (66.2)
No	72	19 (26.4)	53 (73.6)	48	6 (12.5)	42 (87.5)	13	5 (38.5)	8 (61.5)
		*P* = 0.934			*P* = 0.208			*P* = 1.000	
Distant Metastasis									
Yes	41	13 (31.7)	28 (68.3)	20	8 (40.0)	12 (60.0)	13	3 (23.1)	10 (76.9)
No	204	48 (23.5)	156 (76.5)	122	16 (13.1)	106 (86.9)	56	19 (33.9)	37 (66.1)
		*P* = 0.364			*P* = 0.008			*P* = 0.670	
Lauren's histologic type									
Intestinal	154	30 (19.5)	124 (80.5)						
Diffuse	79	27 (34.2)	52 (65.8)						
Mixed	35	16 (47.1)	19 (52.9)						
		*P* = 0.021^a^, 0.002^b^							

a, chi-square test between intestinal- and diffuse-types; b, chi-square test including mixed type.

A total of 268 gastric carcinoma patient samples showed no particular correlation with any clinicopathological features except WHO classification and Lauren's histologic type. *P*<0.05 is considered statistically significant.

The percentage cases with high SP1 expression in intestinal-type gastric cancer was significantly reduced in the distant metastasis group (*P* = 0.008, Table 1). The survival plot supports these results, as the group with the poorest prognosis is also the group with the lowest SP1 expression (*P* = 0.0111, Figure 1C). In contrast, diffuse-type gastric cancer exhibited a negative correlation with SP1 expression; high expression correlated with low survival rates (*P* = 0.0133, Figure 1D). The diffuse type does not appear to be related to any of the unfavorable clinicopathological parameters, as none were statistically significant (Table 1).

The hazard ratios of various clinicopathological factors, including SP1 expression, were estimated using the Cox proportional hazard model. The most significant factors were tumor size and pathological stage in both subtypes, and SP1 expression exhibited a considerable significance in univariate Cox analysis (Table 2). As observed in the Kaplan-Meier curves, the univariate analysis indicated an inverse relationship between SP1 expression and patient survival in intestinal- (hazard ratio  = 0.516, *P* = 0.012) and diffuse-type (hazard ratio  = 2.218, *P* = 0.016) gastric cancer. In the multivariate Cox analysis, the pathological stage retained the highest significance of all of the parameters assessed, whereas tumor size became insignificant in both subtypes (Table 2). Although the overall significance of SP1 expression decreased in multivariate analysis, the hazard ratio indicated a marginally significant correlation between low SP1 expression and overall survival in intestinal-type cancer (hazard ratio  = 0.603, *P* = 0.075).

**Table 2:** Univariate and multivariate survival analyses in intestinal- and diffuse-type gastric carcinoma.

Intestinal type				
Variables	Univariate		Multivariate	
	Hazard ratio (95% confidence interval)	*P*	Hazard ratio (95% confidence interval)	*P*
Age (<60 vs. ≥60)	1.720 (1.059–2.796)	0.029	1.595 (0.919–2.769)	0.097
Sex (F vs. M)	1.010 (0.622–1.637)	0.970		
Size (<5.0 vs. ≥5.0)	3.352 (1.756–6.401)	<0.001	1.274 (0.607–2.674)	0.522
Chemotherapy (No vs. Yes)	1.065 (0.673–1.686)	0.787	0.702 (0.402–1.227)	0.215
Stage (I/II vs. III/IV)	6.329 (3.394–11.80)	<0.001	5.807 (2.764–12.020)	<0.001
SP1: Low (IS<2)	Referent		Referent	
High (IS≥2)	0.516 (0.307–0.867)	0.012	0.603 (0.346–1.051)	0.075

Diffuse type				
Variables	Univariate		Multivariate	
	Hazard ratio (95% confidence interval)	*P*	Hazard ratio (95% confidence interval)	*P*
Age (<60 vs. ≥60)	1.476 (0.834–2.612)	0.181	1.676 (0.906–3.100)	0.100
Sex (F vs. M)	0.914 (0.533–1.568)	0.745		
Size (<5.0 vs. ≥5.0)	2.715 (1.321–5.579)	0.007	1.597 (0.721–3.536)	0.249
Chemotherapy (No vs. Yes)	0.900 (0.505–1.602)	0.720	0.690 (0.371–1.286)	0.243
Stage (I/II vs. III/IV)	5.479 (1.966–15.27)	0.001	4.701 (1.542–14.33)	0.006
SP1: Low (IS<2)	Referent		Referent	
High (IS≥2)	2.218 (1.162–4.234)	0.016	1.733 (0.888–3.382)	0.107

Survival analyses of the effects of various critical clinicopathological factors on SP1 expression were calculated using a Cox proportional hazard model. *P* values <0.05 were considered statistically significant.

### The SP1 expression and classification of 15 gastric cancer cell lines

Based on previous findings [13] and differentiation levels, we divided 15 cell lines into intestinal and diffuse types and classified them as low or high SP1 expressors based on their SP1 levels relative to GAPDH by western blot analysis (Figure 1E). Although all of the diffuse-type cancer cell lines, including SNU5, SNU16, SNU484, SNU601, SNU620, SNU638, SNU668, and KATOIII, belonged to the high-SP1 expressor subtype, several intestinal-type cell lines, such as SNU216, SNU719, and AGS, were low-SP1 expressors, and others, such as MKN1, MKN28, MKN74, and NCI-N87, belonged to the high-expressor subtype (Figure S1A). VEGF levels regulated by SP1 were also examined, but both protein levels failed to exhibit any significant correlation (Figure 1E).

### Alteration of SP1 expression between intestinal- and diffuse-type gastric cancer cells

**Figure 2:**
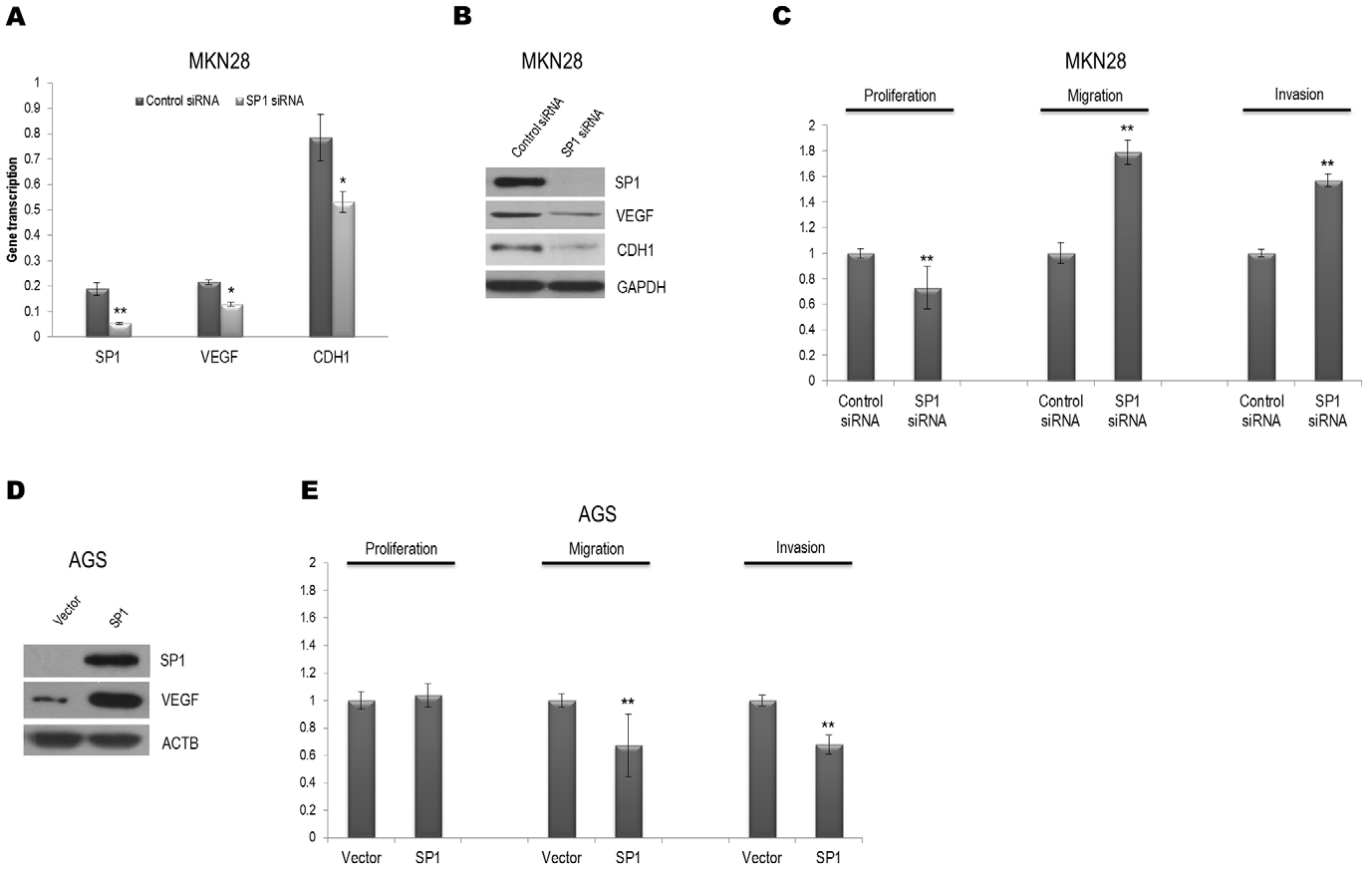
The transfection of the intestinal-type cell lines, MKN28 and AGS, with *SP1* siRNA or *SP1* plasmid controls cell proliferation, cell migration, and invasion. A) MKN28 cells were transfected with *SP1* siRNA or control siRNA. Total mRNA was extracted to determine the expression of endogenous VEGF and CDH1 using qRT-PCR. *HPRT* was used as a control. *, *P*<0.05; **, *P*<0.01. B) Western blot data reveal that *SP1* siRNA causes the knockdown of SP1 and reduces the expression of both VEGF and CDH1 in MKN28. GAPDH was used as a control. C) Cell proliferation, migration, and invasion were assessed after transfection with *SP1* or control siRNA in MKN28. **, *P*<0.01. D) Western blot data demonstrated that SP1 protein levels were increased by transfection with the *SP1* plasmid. ACTB served as the control in AGS. E) Cell proliferation, migration, and invasion assays were performed after transfection with the *SP1* plasmid or a control vector in AGS. **, *P*<0.01.

Because high SP1 expression was correlated with a better prognosis in intestinal-type gastric cancer patients, we hypothesized that changes in the SP1 levels would be reflected in the malignant properties of this type. When endogenous SP1 expression was reduced by siRNA in the high-SP1 expressor intestinal-type cell line, MKN28, the transcription levels of *VEGF* and *CDH1*, both of which harbor GC-rich consensus SP1 binding elements, were decreased (Figure 2A). The protein levels of these genes were also adversely affected by SP1 knockdown (Figure 2B). Although *SP1* siRNA transfection of MKN28 cells inhibited their growth, SP1 knockdown increased their migration and invasion (Figure 2C). These data indicate that the disruption of SP1 is related to VEGF and CDH1 levels and contributes to migration and invasion in intestinal-type gastric carcinoma cells. Moreover, proliferation was decreased in other intestinal-type cells, MKN1 and MKN74. Both migration and invasion were increased in MKN74, but only invasion was augmented in MKN1 (Figure S2A, B). Accordingly, migration and invasion were diminished by transfection with the SP1 expression plasmid in the low-SP1 expressor intestinal-type cell line, AGS (Figure 2D, E).

**Figure 3:**
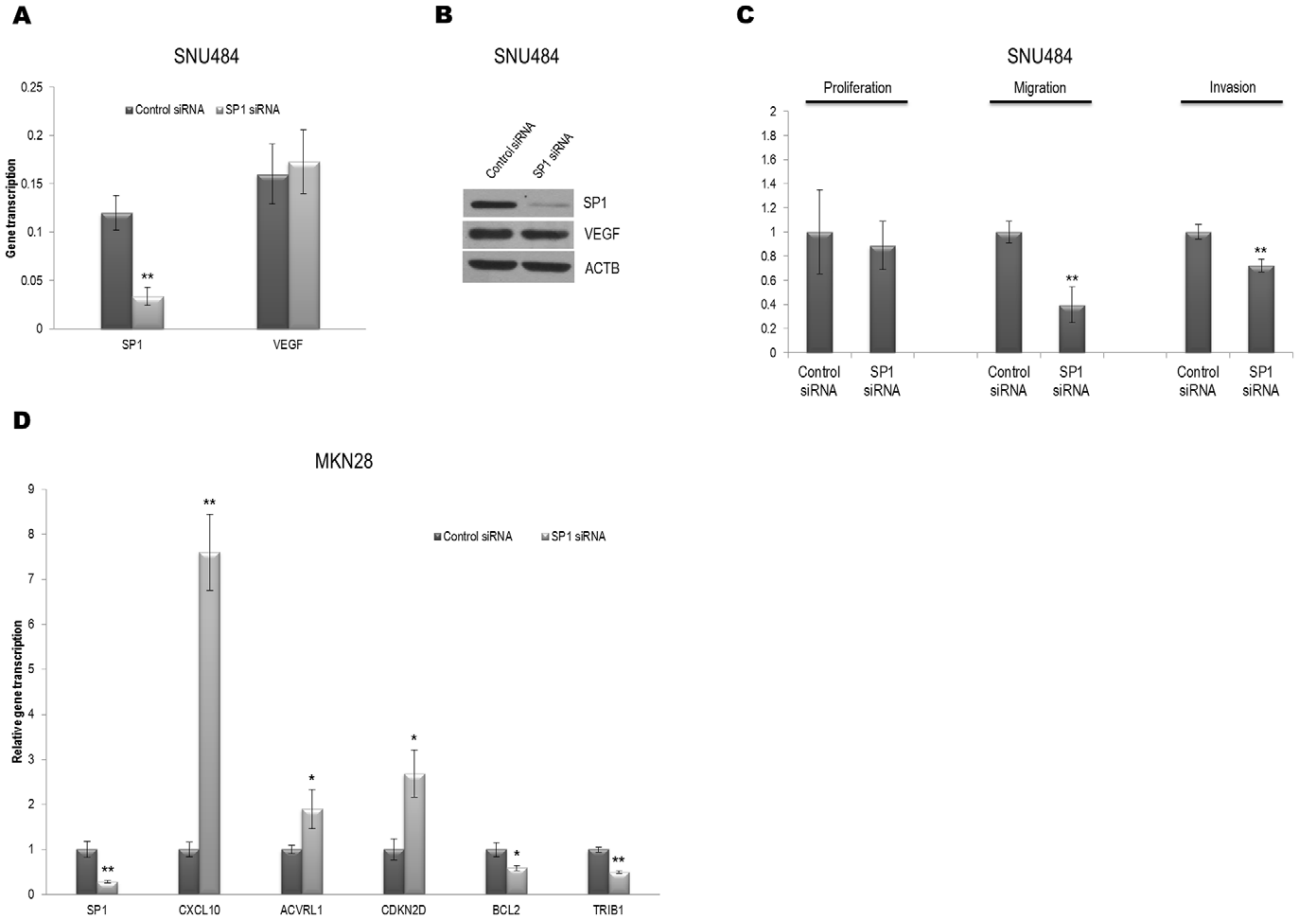
Both cell migration and invasion decrease with decreasing SP1 in SNU484 diffuse-type gastric carcinoma cells, and transcription of genes related to cell proliferation or migration was changed by *SP1* siRNA transfection in MKN28. A) SNU484 cells were transfected with *SP1* siRNA or control siRNA. Total mRNA was extracted to determine the expression of endogenous *VEGF* and *CDH1* by qRT-PCR. *HPRT* was used as a control. **, *P*<0.01. B) Western blot data demonstrate that protein levels of VEGF were not changed by SP1 knockdown in SNU484. ACTB was used as a control. C) Cell proliferation, migration, and invasion were assessed after transfection with *SP1* or control siRNA in SNU484. **, *P*<0.01. D) qRT-PCR of *SP1* along with genes involved in negatively regulating either cell proliferation or cell migration after the transfection of *SP1* siRNA in MKN28 cells demonstrates that SP1 knockdown promotes the elevation of the cell proliferation-inhibiting genes *CXCL10*, *ACVRL1* and *CDKN2D* and the reduction of the cell migration inhibition genes *BCL2* and *TRIB1* at the transcript level. *HPRT* was used as a control. *, *P*<0.05; **, *P*<0.01.

In contrast to the intestinal-type cells, SP1 knockdown by *SP1* siRNA in a diffuse-type cell line, SNU 484, led to decreased migration and invasion, although it did not affect either the mRNA or the protein levels of VEGF or cell proliferation (Figure 3A–C). The CDH1 expression in the SNU484 cells was too low to detect either transcript or protein levels (data not shown). In other diffuse-type cells, SNU638 and SNU668, either migration or invasion were decreased (Figure S2C, D). These results suggest that SP1 expression contributes differently to the regulation of migration and invasion in the 2 types of gastric cancer cells.

### Alteration of SP1 expression influences genes that regulate proliferation and migration in MKN28 cells

To understand how gene expression might be affected by SP1 silencing, we compared SP1 knockdown cells with control siRNA-treated MKN28 cells in an expression array system. The genes in the microarray were divided according to GO terms, and significantly up- or down-regulated genes involved in proliferation and migration were identified (Tables 3 and 4).

**Table 3:** Gene list divided into categories based on gene expression array data of *SP1* siRNA-treated MKN28 cells.

Upregulated genes (fold change >2.5, 277 genes)			
GO term	P-Value	Categories	Gene list
GO:0006952	0.001	defense response	*IL17B CXCL1 IFNB1 IL4 IL6 CXCL10 CXCL9 TLR7 ERAP1 CCL2 CXCL11 CLEC7A BMP2 TFF3 VNN1 RSAD2 IL32 HDAC9*
GO:0006955	0.001	immune response	*TRIM22 GBP4 GBP5 IL17B CXCL1 IFNB1 IL4 IL6 CXCL10 CXCL9 TLR7 ERAP1 CCL2 CXCL11 CLEC7A CBLB VNN1 RSAD2 IL32*
GO:0006954	0.001	inflammatory response	*IL17B CXCL1 IL6 CXCL10 CXCL9 TLR7 CCL2 CXCL11 CLEC7A BMP2 VNN1 HDAC9*
GO:0009611	0.005	response to wounding	*MMRN1 IL17B CXCL1 IL6 CXCL10 CXCL9 TLR7 CCL2 CXCL11 CLEC7A BMP2 VNN1 ACVRL1 HDAC9*
GO:0006935	0.006	chemotaxis	*CXCL1 IL4 IL6 CXCL10 CXCL9 CCL2 CXCL11*
GO:0002376	0.007	immune system process	*TRIM22 CETP GBP4 GBP5 IL17B CXCL1 ID2 IFNB1 IL4 IL6 CXCL10 CXCL9 TLR7 ERAP1 CCL2 CXCL11 CLEC7A CBLB VNN1 RSAD2 IL32 HDAC9*
GO:0008285	0.008	negative regulation of cell proliferation	*CDKN2D CXCL1 IFNB1 IL6 TNFRSF9 PTHLH BDNF BMP2 CTTNBP2 ACVRL1*

Downregulated genes (fold change <0.5, 276 genes)			
GO term	P-Value	Categories	Gene list
GO:0030336	0.002	negative regulation of cell migration	*TRIB1 DLC1 ABHD2 BCL2*
GO:0051271	0.002	negative regulation of cell motion	*TRIB1 DLC1 ABHD2 BCL2*
GO:0014910	0.003	regulation of smooth muscle cell migration	*TRIB1 BCL2*
GO:0014909	0.004	smooth muscle cell migration	*TRIB1 BCL2*
GO:0014812	0.004	muscle cell migration	*TRIB1 BCL2*
GO:0034329	0.006	cell junction assembly	*DLC1 GJA1 BCL2*

**Table 4:** Gene list involved in proliferation and migration based on gene expression array data of *SP1* siRNA-treated MKN28 cells.

Upregulated genes		
Gene symbol	Gene name	Fold change
*CXCL10*	*chemokine (C-X-C motif) ligand 10*	8.35
*ACVRL1*	*activin A receptor type II-like 1*	5.64
*IFNB1*	*interferon, beta 1*	4.64
*CXCL1*	*chemokine (C-X-C motif) ligand 1*	4.61
*ID4*	*inhibitor of DNA binding 4*	3.05
*CDKN2D*	*cyclin-dependent kinase inhibitor 2D*	2.71

Downregulated genes		
Gene symbol	Gene name	Fold change
*ABHD2*	*abhydrolase domain containing 2*	−11.30
*TRIB1*	*tribbles homolog 1*	−2.22
*DLC1*	*deleted in liver cancer 1*	−2.12
*BCL2*	*B-cell CLL/lymphoma 2*	−2.01

Significant changes in the array data were validated by qRT-PCR, and certain genes, such as *CXCL10, ACVRL1*, *CDKN2D*, *BCL2*, and *TRIB1*, enabled us to compare their transcript levels upon *SP1* siRNA transfection into MKN28 cells. These 5 genes were chosen based on their ranks of both fold change and p-values and were targeted for validation. The downregulation of SP1 by siRNA resulted in increased or decreased mRNA levels of these genes as determined by qRT-PCR, which was consistent with the expression array data (Figure 3D).

## Discussion

SP1 overexpression was found in breast cancer, hepatocellular carcinoma, thyroid tumors, and in gastric cancer [14]–[16]. SP1 regulates a variety of cancer associated genes that are related to cell growth, proliferation, angiogenesis, migration, and apoptosis [15], [16]. Our immunohistochemical data are consistent with previous clinicopathological studies demonstrating SP1 overexpression in gastric carcinoma tissues [7]–[9]. In normal tissues and chronic atrophic gastritis, SP1 expression was low. However, as the transformation progressed from intestinal metaplasia to low- and high-grade dysplasia, SP1 expression increased markedly and became high in carcinoma.

Two histological types in gastric cancer should be necessarily considered because intestinal- and diffuse-type have different macroscopic appearance and microscopic growth pattern, reflecting the remarkable difference in their molecular signaling pathway [3]. Our survival data reveal patterns with significant differences when classified according to histological types. In intestinal-type cancer, patients with high SP1 expression had a better prognosis. In contrast, diffuse-type cancer was correlated with a distinct survival pattern in which patient prognosis worsened as SP1 expression increased. Thus, the survival of gastric cancer patients was correlated not only with SP1 expression levels but also with the histological type.

In recent studies, high SP1 and/or VEGF expression were associated with poor survival in human gastric carcinoma [7]–[9]. This observation, however, did not address the difference between intestinal- and diffuse-type cancers. Hence, our results are unique because no other groups have previously included Lauren's classification as an important variable in gastric cancer. Consequently, we divided intestinal- and diffuse-type gastric cancer cell lines into low- or high-SP1 expressor subtypes. Comparisons of cell proliferation, migration, and invasion between non-targeting and *SP1* siRNA-transfected intestinal type cells provided us with possible connections between SP1 and the genes related to these processes. Intestinal type cells with reduced SP1 levels were more capable of migration and invasion than those with high SP1 expression; this result, in part, explains the decreased survival of patients expressing low levels of SP1 in intestinal-type gastric cancer. By contrast, diffuse-type cells with reduced SP1 levels were less capable of migration and invasion. These differences of SP1 role in migration/invasion of intestinal- and diffuse-type gastric cancer show the complexity of SP1-dependent regulation of migration/invasion signaling pathway. This may be related to domain-specific interaction of SP1 with other DNA-bound transcription factor, DNA-independent nuclear factor, SP1 posttranslational modification. Therefore, intestinal- and diffuse- type gastric cancer cells seem to have different migration/invasion signaling pathway and SP1 signaling is dependent on cell context.

Previous studies of SP1 in gastric carcinoma have concentrated on its direct association with VEGF and its involvement in angiogenesis during gastric carcinoma development [8], [17]. However, other mechanisms, such as migration, apoptosis, and cell cycle progression, may have been affected during tumor progression because gastric carcinoma is a disease caused by multiple factors, and SP1 is a transcriptional factor that interacts with many other genes. *CDH1* has a GC-rich DNA region in its promoter that is bound and upregulated by SP1 [18]. A reduction in CDH1 increases cell mobility and promotes tumor cell invasion [19], [20]. These facts are consistent with our observations because cell migration and invasion were increased by the decreased expression of SP1 and CDH1 in intestinal-type cell lines. In contrast, a reduction in SP1 in a diffuse-type cell line did not result in reduced VEGF protein levels, and neither mRNA nor protein levels of CDH1 were detected. It has been reported that approximately 25%–40% of diffuse gastric cancers are caused by *CDH1* inactivation [21], [22]. The difference between the 2 distinct gastric carcinomas can be explained by the involvement of other genes in VEGF and CDH1 regulation or by different types of CDH1 mutations [21]–[23]. Moreover, different genetic alterations were observed in intestinal- and diffuse-type gastric cancers [23].

The loss of SP1 may change the expression of genes involved in cell proliferation and migration. This effect could also explain the poorer survival of low SP1-expressing intestinal-type gastric cancer patients (Figure 1C). The disruption of the basal tissue structure may enhance invasiveness and cancer development in later stages. The association of SP1 with genes mediating proliferation and migration was identified in our results. We observed that genes such as *CXCL10, ACVRL1*, and *CDKN2D*, which are involved in inhibiting cell proliferation, were upregulated by SP1 silencing. CXCL10 inhibits the proliferation of endothelial cells isolated from different tissues [24]. Although its cellular function has long been debated, ACVRL1 expression inhibits endothelial cell proliferation [25], [26]. CDKN2D is periodically expressed during the cell cycle and is maximally induced as cells enter the S phase. When it is constitutively expressed, CDKN2D inhibits CDK4/6 *in vivo* and induces G1-phase arrest [27]. The overall effect of the increased expression of these genes may inhibit tumor cell growth. Although the patterns of SP1 protein levels in gastric cancer cell lines were not directly correlated with the patterns of SP3 and SP4 protein levels (Figure S1B), *CXCL10, ACVRL1*, and *CDKN2D* in MKN28 can also be regulated by SP3 and SP4 in a similar manner to SP1 (data not shown), suggesting that different SP transcription factors can regulate the same gene [28]–[30].

Furthermore, *BCL2* and *TRIB1*, which are involved in cell migration, were found to be downregulated in this study. The promoters of many anti-apoptotic (*BCL2, BCL2L1, etc*.) and pro-apoptotic genes (*BAX, TRAIL, FAS, CASP3, etc*.) contain SP1-binding sites [31]. The anti-apoptotic protein, BCL2, modulates cell adhesive and migratory properties. The overall effect of BCL2 loss is increased cell migration and invasion with decreased adhesion to the extracellular matrix via vitronectin and fibronectin [32]. In contrast, TRIB1 controls vascular smooth muscle cell proliferation and migration [33]. The depletion of TRIB1 leads to an increase in transmigrated cells. These findings are consistent with the results of our study; however, to verify our hypothesis, functional studies are needed.

In summary, we determined that the level of SP1 expression exhibits different patterns in each of Lauren's histological types of human gastric cancers and is closely associated with patient survival. Survival analysis revealed that SP1 levels might become a prognostic biomarker of gastric cancer. In particular, low expression of SP1 is involved in cancer progression and metastasis and may lead to poor prognosis in intestinal-type gastric adenocarcinoma.

## Supporting Information

Figure S1 **The diagram of SP1 expressor and expression of SP family in gastric carcinoma cell lines.** A) Based on these numbers, cells were classified as low or high SP1 expressors as arranged in the diagram. B) SP1, SP3, and SP4 expressions in 10 gastric cancer cell lines were measured using western blot analysis. ACTB was used as the control.
(TIF)

Figure S2 **Cell proliferation, migration, and invasion assay in gastric carcinoma cell lines with** ***SP1*** **siRNA.** A–B) Cell proliferation, migration, and invasion were assessed after transfection with SP1 or control siRNA in intestinal-type cells. *, *P*<0.05; **, *P*<0.01. C–D) Cell proliferation, migration, and invasion were assessed after transfection with SP1 or control siRNA in diffuse-type cells. *, *P*<0.05; **, *P*<0.01.
(TIF)

Table S1 **Primer sequences for qRT-PCR.**
(DOC)

Table S2 **Expression of SP1 in the progression of gastric cancer.**
(DOC)

Materials and Methods S1 (DOC)

## References

[1] Roukos DH, Agnantis NJ, Fatouros M, Kappas AM (2002) (Mini-Review) Gastric Cancer: Introduction, Pathology, Epidemiology. Gastric Breast Cancer 1: 1–3.

[2] Crew KD, Neugut AI (2006) Epidemiology of gastric cancer. World J Gastroenterol 12: 354–362. 16489633 10.3748/wjg.v12.i3.354 PMC4066052

[3] Lauren P (1965) The Two Histological Main Types of Gastric Carcinoma: Diffuse and So-Called Intestinal-Type Carcinoma. An Attempt at a Histo-Clinical Classification. Acta Pathol Microbiol Scand 64: 31–49. 14320675 10.1111/apm.1965.64.1.31

[4] Suske G (1999) The Sp-family of transcription factors. Gene 238: 291–300. 10570957 10.1016/s0378-1119(99)00357-1

[5] Wright C, Angus B, Napier J, Wetherall M, Udagawa Y, et al (1987) Prognostic factors in breast cancer: immunohistochemical staining for SP1 and NCRC 11 related to survival, tumour epidermal growth factor receptor and oestrogen receptor status. J Pathol 153: 325–331. 2828590 10.1002/path.1711530406

[6] Shi Q, Le X, Abbruzzese JL, Peng Z, Qian CN, et al (2001) Constitutive Sp1 activity is essential for differential constitutive expression of vascular endothelial growth factor in human pancreatic adenocarcinoma. Cancer Res 61: 4143–4154. 11358838

[7] Wang L, Wei D, Huang S, Peng Z, Le X, et al (2003) Transcription factor Sp1 expression is a significant predictor of survival in human gastric cancer. Clin Cancer Res 9: 6371–6380. 14695137

[8] Yao JC, Wang L, Wei D, Gong W, Hassan M, et al (2004) Association between expression of transcription factor Sp1 and increased vascular endothelial growth factor expression, advanced stage, and poor survival in patients with resected gastric cancer. Clin Cancer Res 10: 4109–4117. 15217947 10.1158/1078-0432.CCR-03-0628

[9] Zhang J, Zhu ZG, Ji J, Yuan F, Yu YY, et al (2005) Transcription factor Sp1 expression in gastric cancer and its relationship to long-term prognosis. World J Gastroenterol 11: 2213–2217. 15818728 10.3748/wjg.v11.i15.2213 PMC4305801

[10] Lee JH, Kim SH, Wang LH, Choi YL, Kim YC, et al (2007) Clinical significance of CD99 down-regulation in gastric adenocarcinoma. Clin Cancer Res 13: 2584–2591. 17473187 10.1158/1078-0432.CCR-06-1785

[11] Wang LH, Kim SH, Lee JH, Choi YL, Kim YC, et al (2007) Inactivation of SMAD4 tumor suppressor gene during gastric carcinoma progression. Clin Cancer Res 13: 102–110. 17200344 10.1158/1078-0432.CCR-06-1467

[12] Kwon MJ, Oh E, Lee S, Roh MR, Kim SE, et al (2009) Identification of novel reference genes using multiplatform expression data and their validation for quantitative gene expression analysis. PLoS One 4: e6162. 19584937 10.1371/journal.pone.0006162 PMC2703796

[13] Hay R, Park J-G, Gazdar AF (1994) Atlas of human tumor cell lines. San Diego: Academic Press. xii, 486 p.

[14] Deniaud E, Baguet J, Mathieu AL, Pages G, Marvel J, et al (2006) Overexpression of Sp1 transcription factor induces apoptosis. Oncogene 25: 7096–7105. 16715126 10.1038/sj.onc.1209696

[15] Safe S, Abdelrahim M (2005) Sp transcription factor family and its role in cancer. Eur J Cancer 41: 2438–2448. 16209919 10.1016/j.ejca.2005.08.006

[16] Sankpal UT, Goodison S, Abdelrahim M, Basha R (2011) Targeting Sp1 transcription factors in prostate cancer therapy. Med Chem 7: 518–525. 22022994 10.2174/157340611796799203

[17] Wang L, Guan X, Gong W, Yao J, Peng Z, et al (2005) Altered expression of transcription factor Sp1 critically impacts the angiogenic phenotype of human gastric cancer. Clin Exp Metastasis 22: 205–213. 16158248 10.1007/s10585-005-5684-3

[18] Herman JG, Graff JR, Myohanen S, Nelkin BD, Baylin SB (1996) Methylation-specific PCR: a novel PCR assay for methylation status of CpG islands. Proc Natl Acad Sci U S A 93: 9821–9826. 8790415 10.1073/pnas.93.18.9821 PMC38513

[19] Takeichi M (1993) Cadherins in cancer: implications for invasion and metastasis. Curr Opin Cell Biol 5: 806–811. 8240824 10.1016/0955-0674(93)90029-p

[20] Birchmeier W, Behrens J, Weidner KM, Hulsken J, Birchmeier C (1996) Epithelial differentiation and the control of metastasis in carcinomas. Curr Top Microbiol Immunol 213 (Pt 2): 117–135. 10.1007/978-3-642-61109-4_6 9053287

[21] Mayer B, Johnson JP, Leitl F, Jauch KW, Heiss MM, et al (1993) E-cadherin expression in primary and metastatic gastric cancer: down-regulation correlates with cellular dedifferentiation and glandular disintegration. Cancer Res 53: 1690–1695. 8453643

[22] Becker KF, Keller G, Hoefler H (2000) The use of molecular biology in diagnosis and prognosis of gastric cancer. Surg Oncol 9: 5–11. 11525306 10.1016/s0960-7404(00)00016-5

[23] Tahara E (2004) Genetic pathways of two types of gastric cancer. IARC Sci Publ: 327–349. 15055305

[24] Campanella GSV, Colvin RA, Luster AD (2010) CXCL10 Can Inhibit Endothelial Cell Proliferation Independently of CXCR3. PLoS One 5: -. 10.1371/journal.pone.0012700 PMC2938333 20856926

[25] Goumans MJ, Valdimarsdottir G, Itoh S, Rosendahl A, Sideras P, et al (2002) Balancing the activation state of the endothelium via two distinct TGF-beta type I receptors. EMBO J 21: 1743–1753. 11927558 10.1093/emboj/21.7.1743 PMC125949

[26] Lamouille S, Mallet C, Feige JJ, Bailly S (2002) Activin receptor-like kinase 1 is implicated in the maturation phase of angiogenesis. Blood 100: 4495–4501. 12453878 10.1182/blood.V100.13.4495

[27] Hirai H, Roussel MF, Kato JY, Ashmun RA, Sherr CJ (1995) Novel INK4 proteins, p19 and p18, are specific inhibitors of the cyclin D-dependent kinases CDK4 and CDK6. Mol Cell Biol 15: 2672–2681. 7739547 10.1128/mcb.15.5.2672 PMC230497

[28] Jutooru I, Chadalapaka G, Lei P, Safe S (2010) Inhibition of NFkappaB and pancreatic cancer cell and tumor growth by curcumin is dependent on specificity protein down-regulation. J Biol Chem 285: 25332–25344. 20538607 10.1074/jbc.M109.095240 PMC2919096

[29] Chadalapaka G, Jutooru I, Burghardt R, Safe S (2010) Drugs that target specificity proteins downregulate epidermal growth factor receptor in bladder cancer cells. Mol Cancer Res 8: 739–750. 20407012 10.1158/1541-7786.MCR-09-0493 PMC2872686

[30] Abdelrahim M, Baker CH, Abbruzzese JL, Sheikh-Hamad D, Liu S, et al (2007) Regulation of vascular endothelial growth factor receptor-1 expression by specificity proteins 1, 3, and 4 in pancreatic cancer cells. Cancer Res 67: 3286–3294. 17409437 10.1158/0008-5472.CAN-06-3831

[31] Black AR, Black JD, Azizkhan-Clifford J (2001) Sp1 and kruppel-like factor family of transcription factors in cell growth regulation and cancer. J Cell Physiol 188: 143–160. 11424081 10.1002/jcp.1111

[32] Sheibani N, Scheef EA, Dimaio TA, Wang Y, Kondo S, et al (2007) Bcl-2 expression modulates cell adhesion and migration promoting branching of ureteric bud cells. J Cell Physiol 210: 616–625. 17133361 10.1002/jcp.20858

[33] Sung HY, Guan H, Czibula A, King AR, Eder K, et al (2007) Human tribbles-1 controls proliferation and chemotaxis of smooth muscle cells via MAPK signaling pathways. J Biol Chem 282: 18379–18387. 17452330 10.1074/jbc.M610792200 PMC2366084

