# Supplementary figures and images for: Low SP1 Expression Differentially Affects Intestinal-Type Compared with Diffuse-Type Gastric Adenocarcinoma
Source: PLoS One. 2013 Feb 20;8(2):e55522. doi: 10.1371/journal.pone.0055522 (PMC3577840; doi:10.1371/journal.pone.0055522)

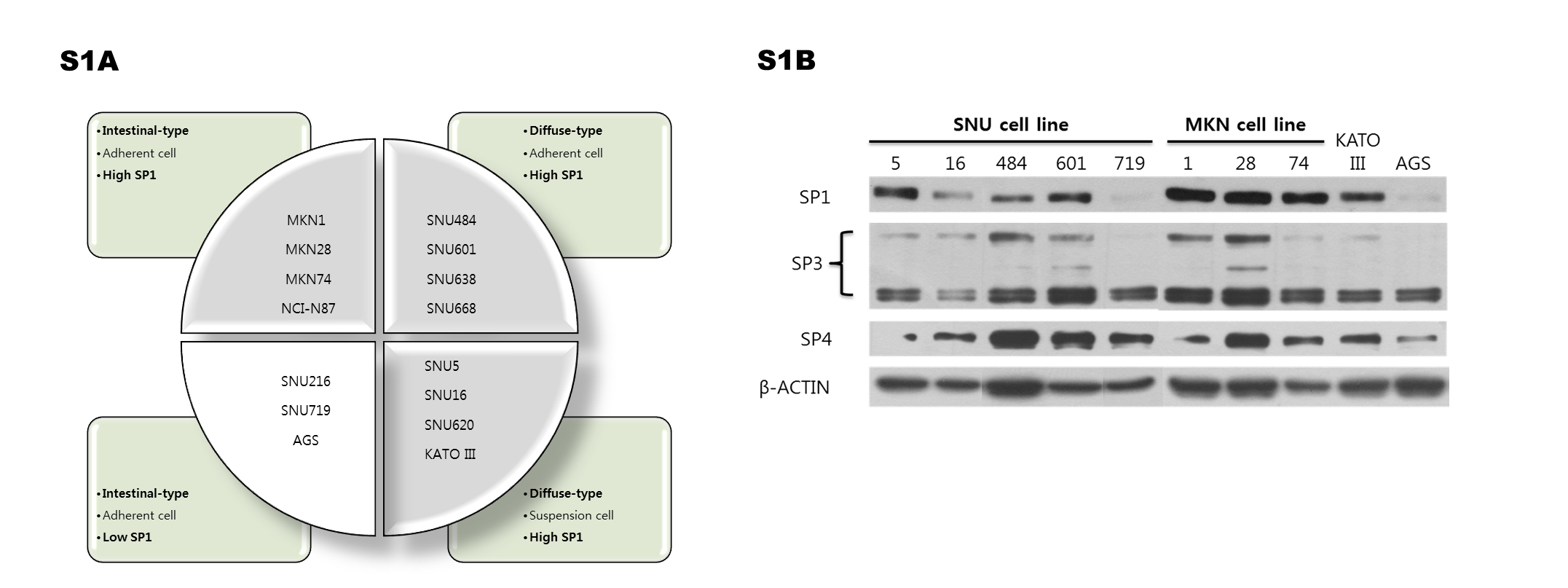

Supplement: Figure S1 — The diagram of SP1 expressor and expression of SP family in gastric carcinoma cell lines. A) Based on these numbers, cells were classified as low or high SP1 expressors as arranged in the diagram. B) SP1, SP3, and SP4 expressions in 10 gastric cancer cell lines were measured using western blot analysis. ACTB was used as the control. (TIF) [file pone.0055522.s001.tif]

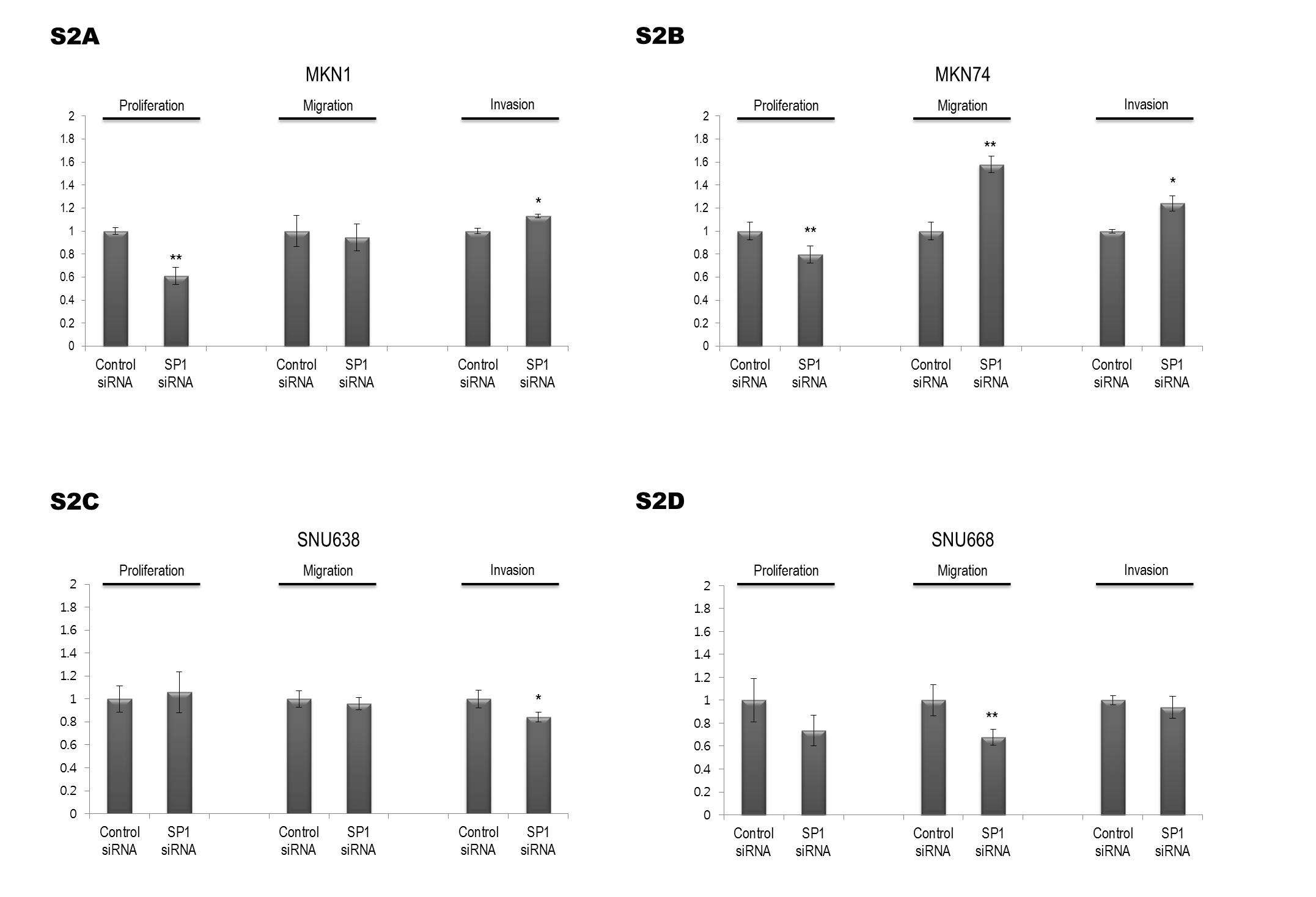

Supplement: Figure S2 — Cell proliferation, migration, and invasion assay in gastric carcinoma cell lines with SP1 siRNA. A–B) Cell proliferation, migration, and invasion were assessed after transfection with SP1 or control siRNA in intestinal-type cells. *, P<0.05; **, P<0.01. C–D) Cell proliferation, migration, and invasion were assessed after transfection with SP1 or control siRNA in diffuse-type cells. *, P<0.05; **, P<0.01. (TIF) [file pone.0055522.s002.tif]
