# Supplementary material for: Low SP1 Expression Differentially Affects Intestinal-Type Compared with Diffuse-Type Gastric Adenocarcinoma
Source: PLoS One. 2013 Feb 20;8(2):e55522. doi: 10.1371/journal.pone.0055522 (PMC3577840; doi:10.1371/journal.pone.0055522)
Supplement: Table S1 — Primer sequences for qRT-PCR. (DOC) [file pone.0055522.s003.doc]

***Table S1* Primer sequences for qRT-PCR**

| **Gene** | **Primer Sequence (5′–3′)** | **UPL No.** |
| --- | --- | --- |
| *SP1* (S) | CTATAGCAAATGCCCCAGGT | # 39 |
| *SP1* (AS) | TCTGGCTGTTTTCTCCTTC |  |
| *VEGF* (S) | GCAGCTTGAGTTAAACGAACG | # 12 |
| *VEGF* (AS) | GGTTCCCGAAACCCTGAG |  |
| *CDH1* (S) | CCCGGGACAACGTTTATTAC | # 35 |
| *CDH1* (AS) | GCTGGCTCAAGTCAAAGTCC |  |
| *CXCL10 (S)* | GAAAGCAGTTAGCAAGGAAAGGT | #34 |
| *CXCL10 (AS)* | GACATATACTCCATGTAGGGAAGTGA |  |
| *ACVRL1* (S) | CGCAATGTGCTGGTCAAG | # 76 |
| *ACVRL1* (AS) | GTTGCCGATGTCCAGGTAAT |  |
| *CDKN2D* (S) | CAGTTTCTTCTGCGCCTCA | # 3 |
| *CDKN2D* (AS) | CTTAAATGCTCTGCCCTTGG |  |
| *BCL2* (S) | AGTACCTGAACCGGCACCT | # 75 |
| *BCL2* (AS) | GGCCGTACAGTTCCACAAA |  |
| *TRIB1* (S) | TACCTGCTGCTGCCCCTA | # 47 |
| *TRIB1* (AS) | TCCTGGTAGTGTTTAATGGGAAA |  |
| *HPRT* (S) | CTCAACTTTAACTGGAAAGAATGTC |  |
| *HPRT* (AS) | TCCTTTTCACCAGCAAGCT |  |
| *HPRT* (probe) | DYXL  TTGCTTTCCTTGGTCAGGCAGTATAATC  BHQ2 |  |

UPL: universal probe library
