# Supplementary material for: Low SP1 Expression Differentially Affects Intestinal-Type Compared with Diffuse-Type Gastric Adenocarcinoma
Source: PLoS One. 2013 Feb 20;8(2):e55522. doi: 10.1371/journal.pone.0055522 (PMC3577840; doi:10.1371/journal.pone.0055522)
Supplement: Table S2 — Expression of SP1 in the progression of gastric cancer. (DOC) [file pone.0055522.s004.doc]

***Table S2* Expression of SP1 in the progression of gastric cancer**

|  | SP1 expression No. (%) | |
| --- | --- | --- |
|  | Low | High |
| NL | 34/38 (89.5%) | 4/38 (10.5%) |
| CAG | 20/24 (83.3 %) | 4/24 (16.7%) |
| IM | 26/38 (68.4 %) | 12/38 (31.6%) |
| LD | 6/12 (50.0%) | 6/12 (50.0%) |
| HD | 7/16 (43.8%) | 9/16 (56.3%) |
| CA | 73/268 (27.2%) | 195/268 (72.8%) |
| TOTAL | 166/396 (41.9%) | 230/396 (58.1%) |

NL, normal; CAG, chronic atrophic gastritis; IM, intestinal metaplasia; LD, low-grade dysplasia; HD, high-grade dysplasia; CA, carcinoma.

Expression of SP1 increases throughout the progression of gastric cancer. Notice that the percentage of low SP1 expression decreases whereas high SP1 expression is increased. *P* < 0.0001 is considered statistically significant.
