# Supplementary material for: Low SP1 Expression Differentially Affects Intestinal-Type Compared with Diffuse-Type Gastric Adenocarcinoma
Source: PLoS One. 2013 Feb 20;8(2):e55522. doi: 10.1371/journal.pone.0055522 (PMC3577840; doi:10.1371/journal.pone.0055522)
Supplement: Materials and Methods S1 — (DOC) [file pone.0055522.s005.doc]

## **Supporting Information Materials and Methods**

## **Patients and tissue specimens**

396 patients with gastric carcinoma and other gastric lesions were analyzed. All patients underwent surgical operations, and none had received chemotherapy or radiotherapy prior to surgery. All patients had well-documented clinical histories and follow-up. Tissue slides were analyzed according to pathological parameters such as tumor size, stage, presence of nodal metastasis, histological grade, depth of invasion, and Lauren’s classification. Ethical approval and permission were obtained from the institutional review board of Chungbuk National University Hospital and Samsung Medical Center. The 268 gastric carcinomas (age: 22–80 years; average age: 56.5 years; 105 female and 163 male cases) comprised 50 early cases and 218 advanced cases. Among these, 30 (11.2%) were classified as tubular well-differentiated adenocarcinoma, 91 (34.0%) as tubular moderately-differentiated adenocarcinoma, 93 (34.7%) as tubular poorly-differentiated adenocarcinoma, 48 (17.9%) as signet ring cell carcinoma, and 6 (2.2%) as mucinous carcinoma. Patient overall survival was followed up to 150 months. Tumor-node-metastasis staging was performed according to the American Joint Committee on Cancer. All archival materials, fixed in 10% neutral-buffered formalin, and embedded in paraffin, were used to generate tissue microarray slides.

## **Preparation of tissue microarray**

To prepare the tissue microarray slides, we punched 3 mm-diameter tissue columns from their original blocks and inserted them into new paraffin blocks with 30 holes each into which the tissue columns were placed. This yielded serially sectioned slides (1 x 3 inches) of gastric carcinoma specimens, enabling analysis of 30 specimens simultaneously with minimum variation during the staining process. Each specimen was round-shaped and 3 mm in diameter, which is sufficient for histopathological analysis. Sections (4 µm) were prepared on silane-coated slides (Sigma, St. Louis, MO).

**Immunohistochemical staining**

Tissue microarray microslides (4 µm) were deparaffinized with xylene, hydrated with a series of diluted alcohol, and immersed in 3% H_2_O_2_ to quench endogenous peroxidase activity. The sections were microwaved in 10 mM citrate buffer for 20 min to retrieve antigen , then incubated with SP1 (PEP2) antibody (sc-59, 1:50 dilution, Santa Cruz Biotechnology, Santa Cruz, CA) for 60 min before rinsing 3 times with wash buffer. The slides were incubated for 20 min at room temperature with anti-rabbit dextran polymer (Envision K4003, DAKO, Glostrup, Denmark) as the secondary antibody. After 3 rinses with distilled water with 0.1% Tween 20, the slides were washed and the chromogen was developed for 5 min with liquid 3,3’-diaminobenzidine (DiNonA, Seoul, Korea), . The slides were counter-stained with Meyer’s hematoxylin, dehydrated, and mounted using Canada balsam for bright-field microscopy.

**Evaluation of results**

Staining intensity was subclassified as follows: 1, weak; 2, moderate; or 3, strong. The positive cells were quantified as a percentage of the total number of epithelial cells and were assigned to 1 of 5 categories: 0, < 5%; 1, 5–25%; 2, 26–50%; 3, 51–75%; and 4, > 75%. The percent positivity of the tumor cells and the staining intensity were then multiplied to generate the immunoreactive score (IS) for each tumor specimen. Each lesion was separately examined and scored by 2 pathologists, and cases with discrepant scores were discussed to reach consensus.

## **Cell lines and whole cell protein extraction**

Fifteen gastric cancer cell lines, SNU5, SNU16, SNU216, SNU484, SNU601, SNU620, SNU638, SNU668, SNU719, MKN1, MKN28, MKN74, KATOIII, AGS, and NCI-N87 (purchased from Korean Cell Line Bank, Seoul, Korea), maintained in RPMI1640 with 10% fetal bovine serum (FBS) and 1% antibiotics were harvested and lysed for 20 min with regular vortexing on ice in RIPA buffer (150 mM NaCl, 50 mM Tris, pH7.6, 10% Glycerol, 0.1% SDS, 1% NP40, 0.5% deoxycholic acid) and protease inhibitors (Roche Diagnostics GmbH, Mannheim, Germany). The lysates were cleared by centrifugation at 14,000 rpm for 15 min at 4°C. Protein concentrations were measured by the BCA assay (Thermo Scientific, Rockford, IL), using bovine serum albumin (BSA) as a control standard.

## ***SP1* siRNA and *SP1* plasmid transfection**

Control or *SP1* specific siRNA oligonucleotides (ON-TARGET plus Smart pool, Thermo Scientific Dharmacon Products, Lafayette, CO) were transfected into SNU484 or MKN28 cells using siLentFect™ Lipid (BIO-RAD, Hercules, CA). At about 30% cell confluency, 10 nM siRNA and 10 µL siLentFect™ Lipid were added to Opti-MEM reduced serum media (Invitrogen Corporation, Carlsbad, CA) to yield a total volume of 1 mL. Cells from triplicate cultures were harvested after 72 h incubation and each was used for cell migration, qRT-PCR, and western blotting assays.

The *SP1* expression plasmid was obtained from Dr. MW Hur (Yonsei University College of Medicine, Seoul, Korea). Twenty-four hours after seeding, AGS cells were transfected with 1 µg of the *SP1* construct in 100-mm culture dishes. Cells were harvested in triplicate after 24 h incubation and each was used for aforementioned experimental purposes.

## **Cell proliferation assay**

1 × 10^4^ siRNA-transfected cells were incubated in triplicate in a 24-well plate for 72 h at 37 ℃. EZ-Cytox solution (50 µL, Daeil Lab Service, Seoul, Korea) was added to each well and incubated for 2 h. The number of surviving cells was measured in a 96-well plate at an optical density of 492 nm on a Sunrise reader (Tecan, Männedorf, Switzerland).

## **Western blot**

The proteins (20-40 µg) from each sample were subjected to 10% SDS/polyacrylamide gel electrophoresis (PAGE) and transferred onto polyvinylidene difluoride (PVDF) membranes (Millipore Corporation, Bedford, MA). Membranes were blocked in 5% non-fat milk in TBS-T buffer for 60 min. Primary antibodies against SP1 (PEP2), SP3 (D20, sc-644, Santa Cruz Biotechnology), SP4 (V20, sc-645, Santa Cruz Biotechnology), VEGF (ab46154, Abcam, Cambridge, MA), and CDH1 (4A2C7 clone, 18-0223, Zymed, Carlsbad, CA) were used separately at 1:2000 dilutions in 5% non-fat milk in TBS-T buffer for 90 min, followed by washing with TBS-T buffer. Membranes were soaked in anti-mouse or rabbit secondary antibody (1:4000) in 5% non-fat milk in TBS-T buffer for 60 min before washing again in TBS-T buffer for 30 min. Both ACTB (C4 clone, sc-47778, Santa Cruz Biotechnology) and GAPDH (FL335 clone, sc-25778, Santa Cruz Biotechnology) were used as controls. The results were visualized with horseradish peroxidase-conjugated secondary antibodies (Thermo Fisher Scientific, Rockford, IL).

**Reference**

1. Kim SH, Kook MC, Shin YK, Park SH, Song HG (2004) Evaluation of antigen retrieval buffer systems. J Mol Histol 35: 409-416.

2. Kim SH, Kook MC, Song HG (2004) Optimal conditions for the retrieval of CD4 and CD8 antigens in formalin-fixed, paraffin-embedded tissues. J Mol Histol 35: 403-408.

3. Kim SH, Shin YK, Lee KM, Lee JS, Yun JH, et al. (2003) An improved protocol of biotinylated tyramine-based immunohistochemistry minimizing nonspecific background staining. J Histochem Cytochem 51: 129-132.
